# Our Journal

**Published:** 1866-12

**Authors:** 


### EDITORIAL AND MISCELLANEOUS.

### OUR JOURNAL.

The present issue makes the tenth number, "New Series," of our Journal. For ten months, under very embarrassing circumstances, the editors and proprietors have labored to reëstablish it upon firm basis, by making such a periodical as the profession of medicine require—a medium of communication for the thoughts and discoveries of physicians, for the discussion of scientific questions amongst medical men, and for giving the medical news of the day. This we have done to the best of our ability, without demanding of patrons pre-payment for subscriptions and advertisements. Now, however, as the volume has advanced nearly to its close, and as the general pecuniary embarrassment that precluded payment at the commencement of the year, has, to some extent, been relieved, we hope and believe that the subscription price of the Journal will be forwarded at once, by mail, at our risk, by those who have received it.

Monthly cash payments, at high rates, to the publisher of the Journal, has well nigh exhausted all the means that can be applied in that way, but by prompt remittance, those indebted to us can, without detriment to themselves, from the small amount each owes, afford the means to relieve us from the pressing demand. Pay up, if you please.

